# Posterior Maxillary Implant Rehabilitation Using Staged Ridge Preservation and Soft‐Tissue Augmentation: A 2‐Year Case Report

**DOI:** 10.1155/crid/3315838

**Published:** 2026-09-06

**Authors:** Nissaf Daouahi, Yosra Gassara, Afef Slim, Chokri Abdellatif

**Affiliations:** ^1^ Department of Fixed Prosthodontics, Faculty of Dentistry, Monastir University, Monastir, Tunisia, um.rnu.tn; ^2^ Department of Surgery, Faculty of Dentistry, Monastir University, Monastir, Tunisia, um.rnu.tn

**Keywords:** alveolar ridge preservation, crestal sinus floor elevation, implant-supported prosthetic rehabilitation, minimally invasive implantology, peri-implant phenotype, soft-tissue augmentation

## Abstract

**Background:**

Rehabilitation of the posterior maxilla is challenging due to sinus pneumatization, limited vertical bone height, and thin peri‐implant soft tissues. Minimally invasive, biologically guided approaches aim to reduce morbidity while ensuring predictable outcomes. This report presents a staged protocol integrating hard‐ and soft‐tissue augmentation to optimize implant site development.

**Case Presentation:**

A 48‐year‐old female presented with a missing maxillary left first molar (#26) and impaired mastication. The left premolars (#24 and #25) were buccopalatally malaligned and exhibited a poor periodontal prognosis. Radiographic examination revealed poor‐prognosis premolars, advanced ridge atrophy, and sinus pneumatization, with a residual bone height < 6 mm. Initial alveolar ridge preservation was performed, but vertical bone gain remained insufficient after 6 months.

**Intervention:**

Following extraction of the premolars, socket preservation with bone grafting and a barrier membrane was performed. Implant placement was performed using a minimally invasive crestal sinus floor elevation with simultaneous grafting, achieving vertical bone augmentation and primary stability. Standard OSSTEM implants (4.5/11 and 4.5/10 mm) were used. After osseointegration, staged peri‐implant soft‐tissue augmentation increased keratinized mucosa width and mucosal thickness to improve tissue stability. The implants were restored with delayed‐loading, screw‐retained metal‐ceramic single crowns following complete soft‐tissue maturation.

**Results:**

The staged protocol achieved stable vertical bone gain, healthy peri‐implant soft tissues, and successful functional and esthetic rehabilitation. These outcomes were maintained at the 2‐year follow‐up after crown delivery, with no biological or prosthetic complications, stable marginal bone levels, a favorable emergence profile, and high patient satisfaction.

**Conclusion:**

Sequential alveolar ridge preservation, minimally invasive crestal sinus elevation, and delayed soft‐tissue augmentation provide a safe, predictable approach for atrophic posterior maxillary sites. This strategy minimizes surgical morbidity while enhancing implant stability, peri‐implant tissue health, and long‐term clinical outcomes.

## 1. Introduction

Implant rehabilitation of the posterior maxilla remains a significant clinical challenge due to sinus pneumatization, progressive alveolar bone resorption, and reduced residual vertical bone height. These anatomical limitations frequently compromise primary implant stability and prosthetically driven positioning, and may negatively influence long‐term treatment outcomes. In cases of insufficient residual bone height, maxillary sinus floor elevation remains the reference standard for vertical augmentation, supported by substantial long‐term evidence demonstrating high survival rates. Although the use of short implants has been proposed as a less invasive alternative to avoid sinus augmentation procedures, this approach remains controversial. Concerns persist regarding the reduced bone‐to‐implant contact surface, altered crown‐to‐implant ratio, and the potential for increased biomechanical loading, particularly in the posterior region where occlusal forces are higher. Furthermore, evidence on their long‐term predictability remains heterogeneous, especially in severely atrophic maxillae. Consequently, careful case selection and a thorough understanding of biomechanical and anatomical limitations are essential when considering short implants as a treatment option [1–5].

Advances in surgical techniques, biomaterials, and implant design have expanded the scope of conventional‐length implants even in atrophic sites. In this report, conventional‐length implants are defined as fixtures ≥ 10 mm in length, as opposed to short implants (typically ≤ 6–8 mm), in accordance with commonly used classifications. Alveolar ridge preservation (ARP) and augmentation strategies effectively mitigate postextraction bone loss, optimize three‐dimensional bone architecture, and enable more predictable implant placement. Minimally invasive transcrestal sinus elevation now provides a safe and efficient alternative to the lateral window approach. Innovations such as hydraulic or osseodensification‐assisted elevation, coupled with modern instruments including piezoelectric devices and specialized drills, have enhanced precision, reduced surgical trauma, and maintained high clinical success [6–11].

The peri‐implant soft‐tissue phenotype constitutes an equally decisive determinant of implant longevity. Key parameters—keratinized mucosa width, mucosal thickness, and supracrestal tissue height, first conceptualized during the 2017 World Workshop and subsequently translated into implantology—have been shown to exert a pivotal influence on mucosal stability, curbing recession and plaque‐induced inflammation, restraining marginal bone loss, and optimizing esthetic integration [12–15].

The timing of soft‐tissue augmentation remains nuanced. Simultaneous grafting at implant placement can minimize interventions and patient morbidity, whereas staged procedures allow more precise management of tissue maturation, vascularization, and esthetic contouring. Personalized selection based on local anatomy, tissue phenotype, and the extent of hard‐tissue reconstruction is critical to maximize predictability and long‐term success [16].

## 2. Objective

The objective of this case report is to document a staged, minimally invasive treatment protocol for implant‐supported rehabilitation of the atrophic posterior maxilla, combining ARP, transcrestal sinus floor elevation, delayed implant placement, and secondary soft‐tissue augmentation. This sequential approach was designed to achieve predictable primary stability and long‐term survival of conventional‐length implants (4.5 × 11 and 4.5 × 10 mm) despite severe anatomical limitations, thereby offering a viable alternative to more invasive techniques.

## 3. Clinical Presentation

**Figure 1:**
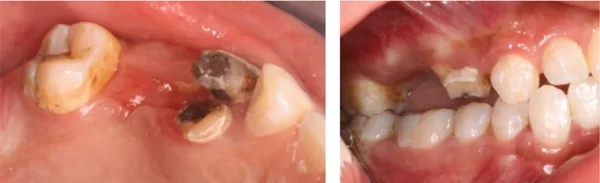
Clinical view of the initial situation.

A 48‐year‐old female presented with impaired mastication in the left maxilla and a request for replacement of the missing maxillary first molar (#26). She also reported increasing functional discomfort. Clinical examination revealed an edentulous site at #26. The premolars (#24 and #25) were buccopalatally malaligned and exhibited a poor prognosis. Periodontal examination established a diagnosis of generalized Stage III–IV periodontitis affecting Teeth #24 and #25, with probing depths ranging from 7 to 9 mm, Grade II mobility, and bleeding on probing (Figure 1).

**Figure 2:**
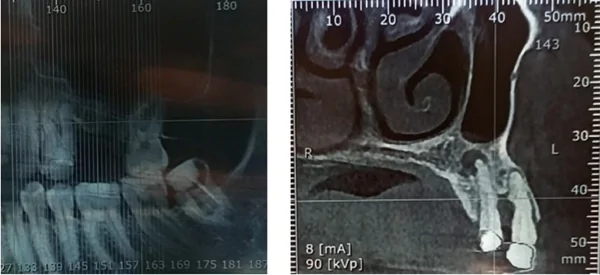
Radiographic examination of the initial situation.

Cone‐beam computed tomography (CBCT) demonstrated combined vertical and horizontal alveolar bone loss affecting both premolars, with marked buccal and interradicular defects consistent with advanced chronic periodontitis. In the posterior maxilla, sinus pneumatization further reduced residual bone height at the first molar site to less than 6 mm, precluding conventional implant placement. Given the severe hard‐tissue loss, unfavorable tooth position, and poor restorative prognosis, both premolars were deemed nonrestorable and indicated for extraction (Figure 2). Clinical examination revealed a thin peri‐implant soft‐tissue phenotype, indicating the need for augmentation as part of a staged, multidisciplinary approach.

The treatment plan included the extraction of nonrestorable premolars, ARP, and delayed implant placement following radiographic reassessment of bone volume. This staged strategy is supported by evidence showing improved hard‐ and soft‐tissue stability in posterior maxillary implant rehabilitation. ARP reduces postextraction resorption and facilitates predictable delayed implant placement [17]. Evaluation of peri‐implant tissue thickness after healing guides the indication for soft‐tissue grafting and improves long‐term peri‐implant stability, particularly in thin biotypes. Both simultaneous and staged connective tissue grafting effectively increase keratinized tissue width and mucosal thickness, allowing flexible timing based on clinical conditions [18, 19].

Following extraction of Teeth #24 and #25, ARP was performed using a mucoperiosteal flap technique with minimal flap reflection to preserve soft‐tissue architecture and facilitate graft containment. A midcrestal incision confined to the edentulous and extraction sites was performed without vertical releasing incisions to limit soft‐tissue trauma and maintain vascular supply to the flap margins, consistent with the clinical figures. The site was managed with primary closure to ensure complete coverage of the grafted socket and to optimize uneventful healing.

The socket was grafted using a xenograft bone substitute (deproteinized bovine bone mineral; Bio‐Oss, Geistlich Pharma AG, Wolhusen, Switzerland) hydrated and mixed with the patient′s autologous blood before placement to optimize handling, adaptation, and graft stability, thereby maintaining ridge volume and reducing postextraction resorption. The grafted site was covered with a resorbable collagen membrane (Bio‐Gide, Geistlich Pharma AG, Wolhusen, Switzerland) to provide barrier function and stabilize the grafted material. Primary closure was achieved using nonresorbable monofilament sutures.

**Figure 3:**
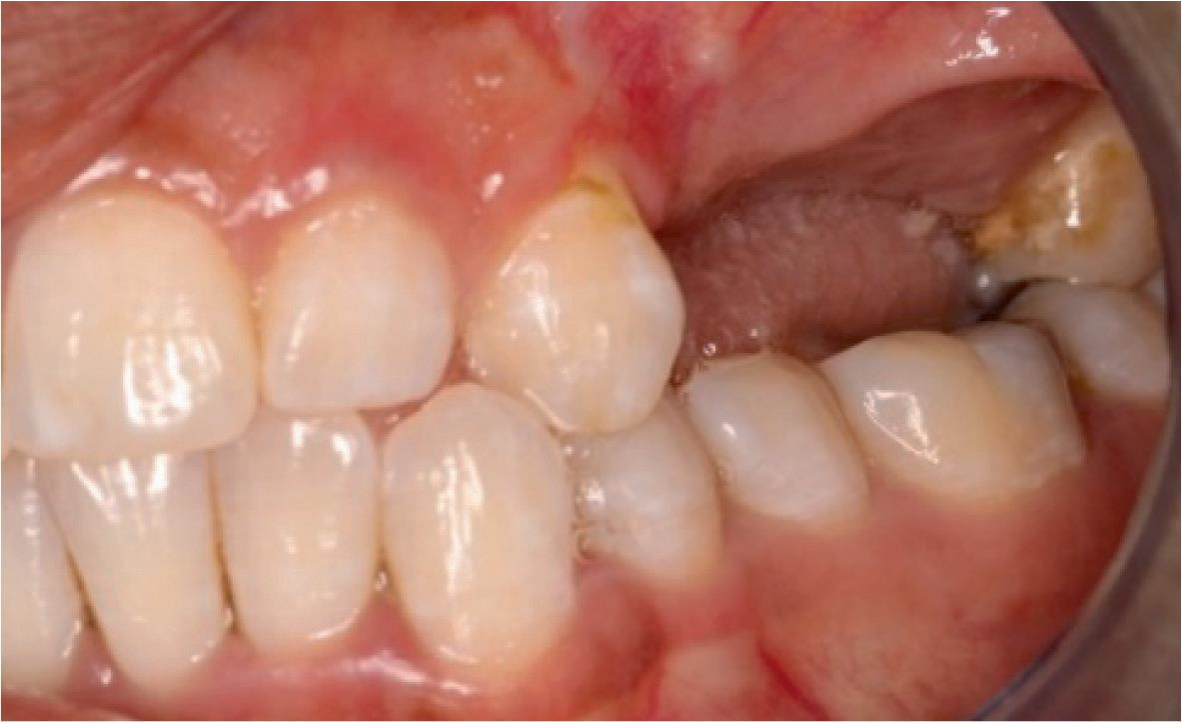
Clinical view after the healing period following crestal ridge reconstruction and socket preservation.

**Figure 4:**
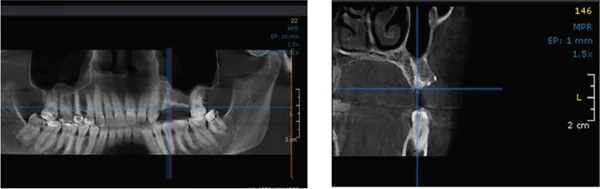
Radiographic examination after extraction and socket preservation, demonstrating mature regenerated bone at the 6‐month healing period.

**Figure 5:**
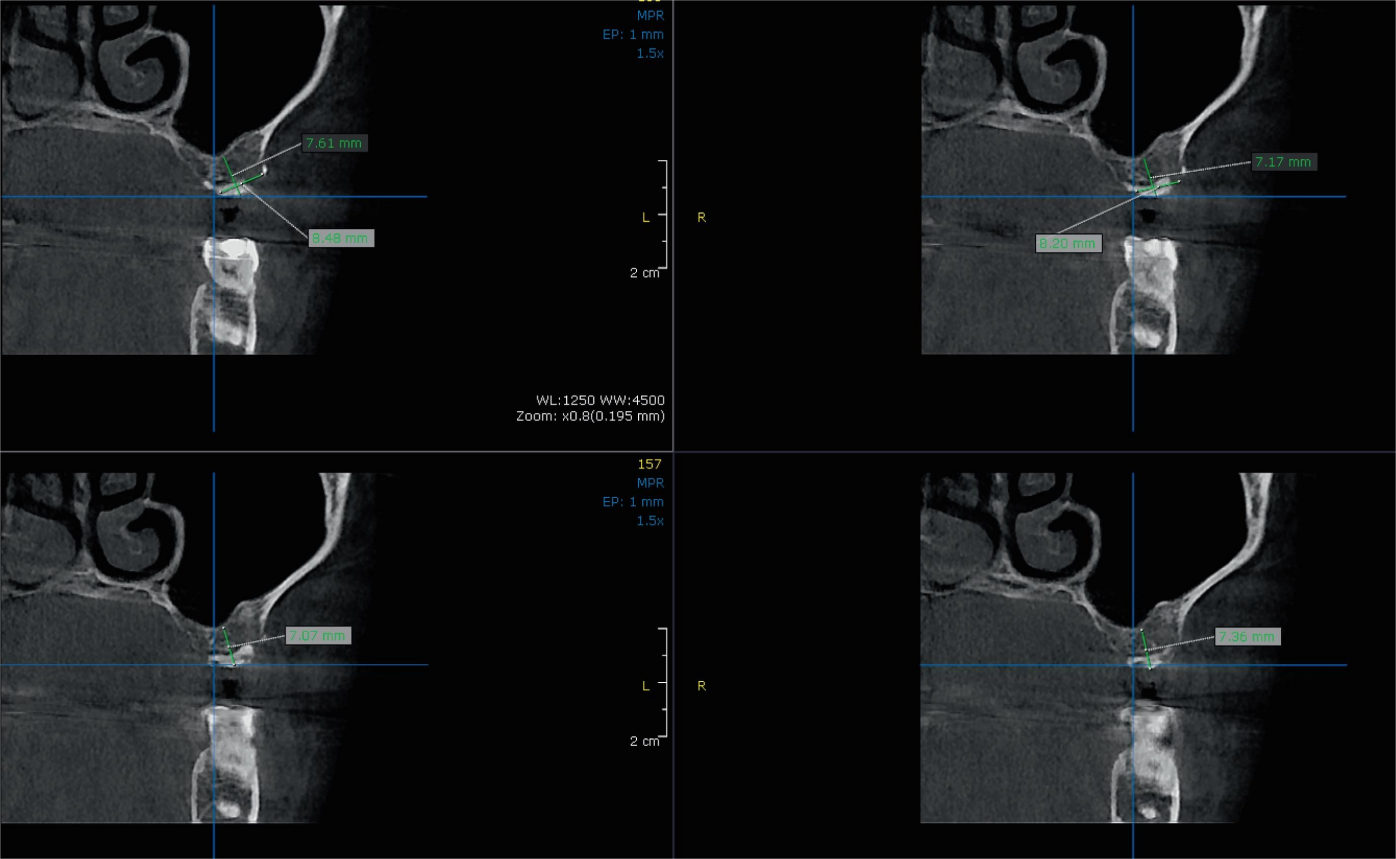
Assessment of alveolar bone support using cross‐sectional CBCT slices.

Six months after ARP, radiographic evaluation demonstrated mature regenerated bone. However, sinus pneumatization required sinus floor elevation to achieve adequate vertical bone height for implant placement (Figures 3, 4, and 5).

**Figure 6:**
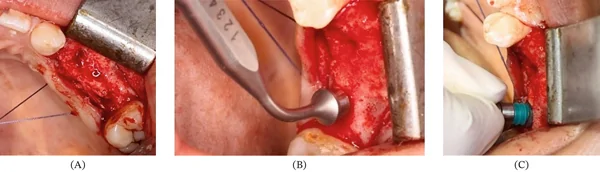
Crestal sinus floor elevation. (A) Midcrestal incision and flap elevation exposing the alveolar ridge; (B) sequential osteotomy preparation with calibrated drills; and (C) transcrestal approach to the sinus floor before membrane elevation.

Crestal sinus floor elevation was performed simultaneously with the placement of two standard implants (OSSTEM, Osstem Implant Co., Busan, South Korea): 4.5 × 11 and 4.5 × 10 mm. They were selected according to residual bone height, sinus anatomy, and ridge morphology. The CAS‐Kit (Osstem Implant Co., Busan, South Korea) was used to perform a minimally invasive transcrestal sinus elevation. After antisepsis with 0.12% chlorhexidine and administration of local anesthesia (buccal and palatal infiltration), a midcrestal incision was made, and a full‐thickness mucoperiosteal flap was elevated to expose the alveolar ridge (Figure 6A–C).

**Figure 7:**
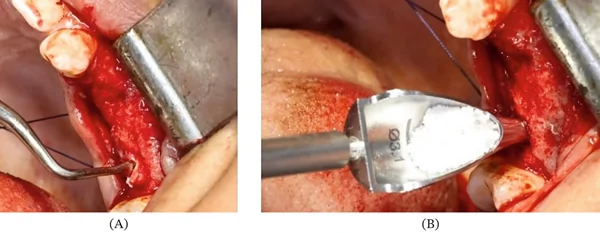
Grafted bone positioned between the implant apex and the sinus membrane. (A) Hydraulic elevation of the Schneiderian membrane; (B) placement of particulate xenograft beneath the elevated membrane.

**Figure 8:**
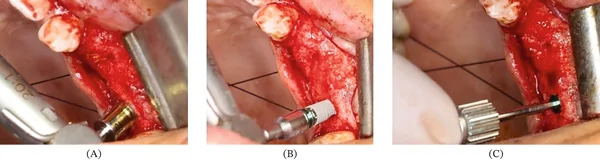
Precise positioning of implants. (A–C) Sequential implant insertion steps at the two osteotomy sites.

**Figure 9:**
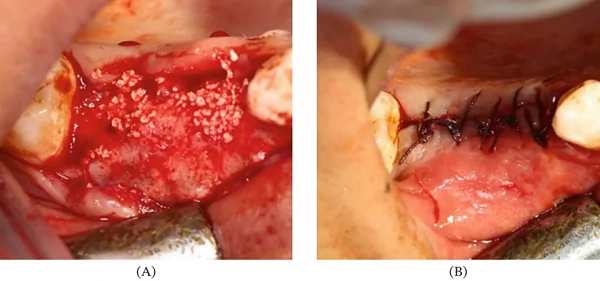
(A) Occlusal view of the surgical site after placement of particulate bone graft material within the alveolar defect. (B) Primary wound closure achieved with interrupted sutures.

**Figure 10:**
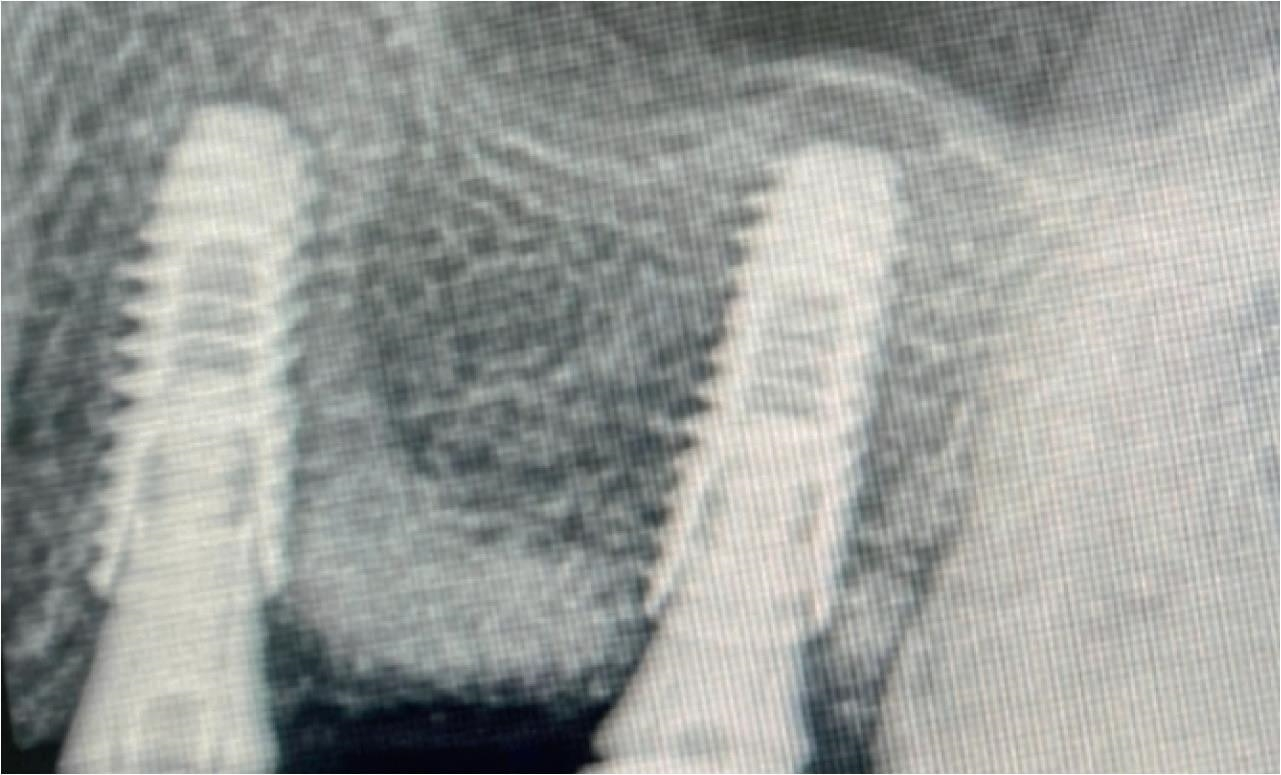
Radiograph showing successful osseointegration of the implant after the healing period, with healing abutments in place.

Sequential osteotomy preparation was performed using calibrated drills with depth stoppers. A 2.8‐mm round bur was used to carefully approach the sinus cortical floor, minimizing the risk of Schneiderian membrane perforation. The membrane was elevated using a hydraulic pressure technique, allowing an atraumatic lift of approximately 3–4 mm and controlled augmentation of the subantral space. Particulate bone graft material consisting of a xenograft (Bio‐Oss, Geistlich Pharma AG, Wolhusen, Switzerland) was introduced beneath the elevated Schneiderian membrane to support space maintenance and promote bone regeneration (Figure 7A,B). Implants were placed with a controlled insertion torque of 35 Ncm, ensuring adequate primary stability (Figure 8A–C). The flap was repositioned and closed with a tension‐free wound closure to optimize soft‐tissue healing and minimize postoperative complications (Figures 8 and 9). Radiographic follow‐up at 6 months confirmed successful osseointegration with stable crestal bone levels and no signs of peri‐implant radiolucency (Figure 10).

**Figure 11:**
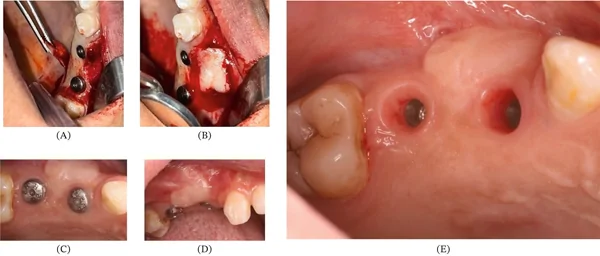
Management and augmentation of soft tissues. (A) Harvesting of the connective tissue graft from the palatal donor site; (B) graft adaptation at the recipient site; (C) healing abutments in place after augmentation; (D) clinical view of the peri‐implant mucosa; (E) Occlusal clinical view showing the emergence profile of the peri‐implant soft tissue.

Four months after implant placement, following confirmation of successful osseointegration, staged peri‐implant soft‐tissue augmentation was performed to improve the peri‐implant phenotype (Figure 11A–D). A connective tissue graft was harvested from the palatal donor site (contralateral region) and placed to increase keratinized mucosa width and soft‐tissue thickness. This procedure was performed before the prosthetic phase. The graft was stabilized with tension‐free wound closure to ensure optimal integration and vascularization. The augmentation resulted in improved peri‐implant tissue stability, facilitated oral hygiene maintenance, and enhanced the emergence profile.

Peri‐implant soft tissues were allowed to mature for approximately 3 months, in accordance with evidence indicating that functional epithelial sealing and connective tissue remodeling occur within 8–12 weeks, whereas complete maturation and dimensional stability may extend up to 3–6 months depending on biotype and augmentation procedures. This healing period was respected before prosthetic rehabilitation to ensure stable soft‐tissue architecture and optimal emergence profile development.

**Figure 12:**
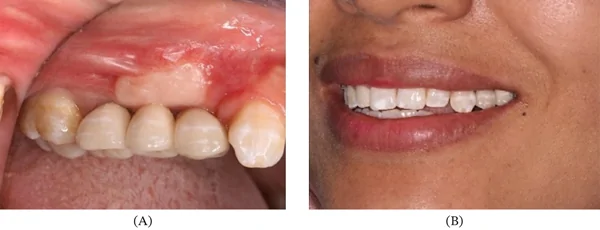
(A) Intraoral lateral view of the peri‐implant soft tissue and adjacent dentition. (B) Extraoral frontal smile view demonstrating the esthetic integration of the implant‐supported restoration.

**Figure 13:**
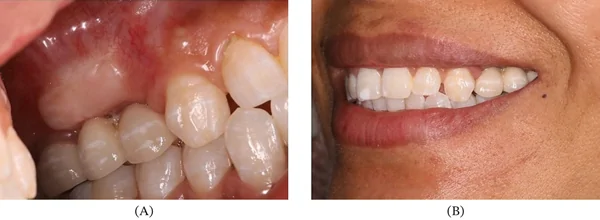
Clinical outcome at the 2‐year follow‐up.

The prosthetic phase consisted of delayed‐loading, metal‐ceramic bridge fabricated after complete soft‐tissue maturation. Screw retention was selected to preserve retrievability and facilitate peri‐implant tissue maintenance. The prosthetic phase was initiated only after complete soft‐tissue maturation and clinical stability were confirmed, allowing precise prosthetic contouring, improved hygiene access, and long‐term peri‐implant tissue stability (Figures 12 and 13).

At the 2‐year follow‐up, assessed from the date of definitive crown delivery, both implants remained clinically and radiographically stable, with no biological complications (no peri‐implant mucositis, suppuration, or radiographic peri‐implant radiolucency) and no prosthetic complications (no screw loosening, ceramic chipping, or loss of retention). Peri‐implant soft tissues showed adequate keratinized mucosa width, healthy coloration, a well‐contoured emergence profile, and a stable esthetic outcome consistent with the adjacent dentition (Figure 13).

## 4. Discussion

In the posterior maxilla, tooth extraction and prolonged edentulism initiate progressive alveolar ridge remodeling, characterized by substantial horizontal and vertical bone resorption and, frequently, concomitant pneumatization of the maxillary sinus. This combination may markedly compromise the available three‐dimensional bone volume for implant placement, particularly in the molar region. In the absence of ridge‐preservation or augmentation procedures, postextraction physiological remodeling can result in pronounced dimensional alterations, with reported reductions of up to approximately 2.0 mm in vertical ridge height and 2.7–3.6 mm in horizontal ridge width within the first 6 months after extraction [20–22].

Implant‐prosthetic rehabilitation of the posterior edentulous maxilla is often limited by inadequate residual bone volume due to alveolar ridge resorption and maxillary sinus pneumatization, which frequently necessitates bone augmentation procedures before or in conjunction with implant placement. Socket preservation has been shown to reduce sinus pneumatization and the subsequent need for extensive sinus floor elevation procedures in the posterior maxilla, which supports the initial step of our staged protocol [23].

ARP, achieved by placing bone graft material and/or membranes immediately after tooth extraction, aims to reduce postextraction ridge remodeling. Clinical studies and meta‐analyses have demonstrated that ARP in the posterior maxilla reduces vertical bone loss and limits maxillary sinus pneumatization compared with spontaneous healing. As a result, greater residual bone height is often maintained at the time of implant placement, decreasing the need for more invasive sinus augmentation procedures. For example, alveolar ridge height is significantly greater in ARP‐treated posterior maxillary sites at 6 months postextraction, often maintaining ~7.3 mm of residual bone height versus ~4.8 mm in nongrafted controls, reducing the incidence of advanced sinus augmentation procedures at implant placement [24].

Evidence from systematic reviews indicates that ARP effectively limits maxillary sinus pneumatization and maintains vertical ridge height compared with spontaneous healing, thereby improving site conditions for implant placement in the posterior maxilla. By preserving alveolar dimensions and ridge morphology, ARP contributes to improved surgical predictability and may reduce the need for extensive regenerative procedures [23].

When residual vertical bone remains insufficient, maxillary sinus floor elevation is required to achieve adequate bone height for primary implant stability. The crestal (transcrestal) approach is recognized as a minimally invasive alternative to the lateral window technique, particularly in sites with moderate residual bone height (approximately 3–7 mm), thereby reducing surgical morbidity, shortening treatment time, and accelerating postoperative recovery [25].

Recent randomized controlled trials and meta‐analyses demonstrate that crestal sinus elevation techniques—including hydraulic and osseodensification‐assisted methods—achieve predictable vertical bone gain of approximately 3–4 mm, high implant survival rates (94%–100%), and fewer complications compared with lateral approaches. Procedure selection and graft stability are influenced by anatomical variables such as residual bone height, sinus width, Schneiderian membrane integrity, and bone density. In conjunction with prior ARP, crestal sinus elevation enables predictable reconstruction of atrophic posterior maxillary sites by optimizing residual ridge architecture, supporting implant stability, and improving long‐term functional and esthetic outcomes [26–28].

In cases of severe vertical deficiency where primary implant stability cannot be achieved, a staged crestal sinus elevation approach with delayed implant placement is clinically indicated, as it allows adequate maturation of the grafted bone before implant insertion, thereby reducing the risk of inadequate primary stability and improving the predictability of osseointegration; this staged approach was therefore adopted in the present case.

Peri‐implant soft‐tissue characteristics are key to long‐term implant stability. Techniques such as free gingival grafts, connective tissue grafts, and collagen‐based substitutes increase keratinized mucosa and tissue thickness, helping to maintain peri‐implant health and reduce bone loss, inflammation, and recession [29, 30].

The three‐dimensional soft‐ and hard‐tissue configuration surrounding dental implants—collectively defined as the peri‐implant phenotype—includes keratinized mucosa width, mucosal thickness, supracrestal tissue height, and peri‐implant bone thickness. Derived from the 2017 classification of periodontal and peri‐implant conditions, this concept emphasizes the critical role of peri‐implant tissue dimensions in achieving implant stability, esthetic integration, and long‐term biological success. Emerging clinical and histologic evidence indicates that a favorable peri‐implant phenotype is associated with reduced inflammatory response, limited marginal bone loss, and decreased incidence of peri‐implant soft‐tissue recession [15, 30–32].

Peri‐implant soft‐tissue augmentation aimed at enhancing keratinized mucosa width and mucosal thickness has been systematically reviewed and has demonstrated benefits for peri‐implant health, supporting the rationale for delayed soft‐tissue grafting upon osseointegration [33].

The timing of peri‐implant soft‐tissue augmentation remains a subject of clinical debate. Simultaneous grafting at implant placement is often proposed in sites with a thin peri‐implant phenotype or limited keratinized mucosa to reduce the number of surgical interventions. In contrast, a staged approach performed after implant osseointegration allows for a more accurate assessment of peri‐implant tissue deficiencies, improved graft vascularization, and enhanced control of soft‐tissue contours. Current clinical evidence indicates that delayed soft‐tissue augmentation achieves peri‐implant tissue thickness and long‐term stability comparable with, and in some instances exceeding, simultaneous procedures, particularly when connective tissue grafts or validated soft‐tissue substitutes are employed [34, 35].

In this case, soft‐tissue augmentation was deliberately staged after implant osseointegration because of unfavorable initial soft‐tissue conditions. This approach is supported by evidence showing that, particularly in complex sites, delayed augmentation may provide predictable and stable gains in mucosal thickness and keratinized tissue, comparable with those achieved with connective tissue grafts [19, 36–38].

## 5. Conclusion

A staged approach in the posterior maxilla—combining ARP, minimally invasive crestal sinus floor elevation, and delayed soft‐tissue augmentation—optimizes both hard‐ and soft‐tissue architecture while minimizing surgical morbidity. By respecting appropriate healing intervals, this protocol enhances peri‐implant tissue stability, supports predictable implant osseointegration, and achieves favorable esthetic and functional outcomes. Current evidence underscores that such biologically guided, sequential strategies represent a reliable framework for managing atrophic posterior maxillary sites in contemporary implantology.

## Author Contributions

**Nissaf Daouahi:** conceptualization, investigation, methodology, project administration, writing – original draft, writing – review and editing. **Yosra Gassara:** data curation, investigation, visualization, writing – original draft (literature review, figure preparation). **Afef Slim:** investigation, methodology, surgery (performing procedures), resources. **Chokri Abdellatif:** investigation, methodology, surgery (performing procedures), supervision, writing – review and editing.

## Funding

No funding was received for this manuscript.

## Disclosure

All authors have read and approved the final version of the manuscript. The corresponding author (manuscript guarantor) had full access to all data in this study and takes full responsibility for the integrity and accuracy of the data and analysis.

## Ethics Statement

This retrospective case report was conducted in accordance with institutional ethical standards. Formal Institutional Review Board (IRB) approval was not required for this single, anonymized case report, in accordance with institutional policy exempting retrospective single‐patient case reports involving routine clinical care from formal ethics committee review. Written informed consent for the clinical procedures and for publication of anonymized clinical data and images was obtained from the patient, and consent for formal research ethics review was waived due to the retrospective, single‐case design. No personal identifiers or details that could reveal the patient′s identity are included.

## Conflicts of Interest

The authors declare no conflicts of interest.

## Supporting information


**Supporting Information** Additional supporting information can be found online in the Supporting Information section. CARE checklist (CAse REport guidelines – 2013).

## Data Availability

The authors confirm that the data supporting the findings of this work are available with the corresponding author.
