# Supplementary material for: Posterior Maxillary Implant Rehabilitation Using Staged Ridge Preservation and Soft‐Tissue Augmentation: A 2‐Year Case Report
Source: Case Rep Dent. 2026 Sep 6;2026:3315838. doi: 10.1155/crid/3315838 (PMC13547594; doi:10.1155/crid/3315838)
Supplement: Supplementary file 1 [file CRID-2026-3315838-s001.docx]

# **CARE Checklist**

*(CAse REport Guidelines – 2013)*

**Manuscript 3315838 (Revised Version) – “Posterior Maxillary Implant Rehabilitation Using Staged Ridge Preservation and Soft-Tissue Augmentation: A 2-Year Case Report”**

*Case Reports in Dentistry*

| **Topic** | **Item** | **Reported on Page/Section** |
| --- | --- | --- |
| **Title** | 1. The area of focus (e.g., new diagnosis, unexpected outcome, therapeutic/management challenge, unusual presentation) and the word “case report” should appear in the title. | Title page, lines 1–2 – “Posterior Maxillary Implant Rehabilitation Using Staged Ridge Preservation and Soft-Tissue Augmentation: A 2-Year Case Report.” |
| **Key Words** | 2. Two to five key words that identify diagnoses or interventions in this case report, including “case report.” | Abstract, lines 37–38 – Keywords: Minimally invasive implantology; Alveolar ridge preservation; Crestal sinus floor elevation; Soft-tissue augmentation; Peri-implant phenotype; Implant-supported prosthetic rehabilitation. (“Case report” is reflected in the title and article type.) |
| **Abstract** | 3a. Introduction – What is unique about this case and what does it add to the scientific literature? | Abstract, Background, lines 13–16 – challenges of posterior maxillary rehabilitation and rationale for a staged, biologically guided protocol. |
|  | 3b. Main symptoms/important clinical findings. | Abstract, Case Presentation, lines 17–21 – missing #26, malaligned/poor-prognosis premolars #24–#25, ridge atrophy, sinus pneumatization, residual bone height <6 mm. |
|  | 3c. Main diagnoses, therapeutic interventions, and outcomes. | Abstract, Intervention/Results, lines 22–32 – socket preservation, minimally invasive crestal sinus elevation with simultaneous implant placement, staged peri-implant soft-tissue augmentation, delayed-loading screw-retained crowns; stable outcomes maintained at 2-year follow-up with no complications. |
|  | 3d. Conclusion – What is the main “take-away” lesson from this case? | Abstract, Conclusion, lines 33–36 – sequential alveolar ridge preservation, minimally invasive crestal sinus elevation, and delayed soft-tissue augmentation provide a safe, predictable approach for atrophic posterior maxillary sites. |
| **Introduction** | 4. One or two paragraphs summarizing why this case is unique, with a brief review of relevant literature. | Introduction, lines 39–84 – background on posterior maxillary implant rehabilitation, sinus pneumatization, short- vs. conventional-length implants, peri-implant phenotype, timing of soft-tissue augmentation, and explicit study Objective (lines 77–84). |
| **Patient Information** | 5a. De-identified patient-specific information. | Clinical Presentation, lines 86–87 – 48-year-old female; no identifying details included (see also Declarations – Ethics Approval and Consent, lines 272–280, confirming no personal identifiers are disclosed). |
|  | 5b. Primary concerns and symptoms of the patient. | Clinical Presentation, lines 86–88 – impaired mastication in the left maxilla, missing #26, and increasing functional discomfort. |
|  | 5c. Medical, family, and psychosocial history, including relevant genetic information. | Not applicable/contributory to this localized dental case; no relevant systemic, family, or genetic history is reported in the Clinical Presentation section (lines 85–101). |
|  | 5d. Relevant past interventions and their outcomes. | Clinical Presentation, lines 89–99 – prior condition of teeth #24/#25 (stage III–IV periodontitis, non-restorable) described; no prior implant or grafting interventions at this site. |
| **Clinical Findings** | 6. Describe relevant physical examination (PE) and other clinical findings. | Clinical Presentation, lines 88–101 – clinical exam findings (buccopalatal malalignment, probing depths 7–9 mm, grade II mobility, bleeding on probing) and CBCT findings (combined vertical/horizontal bone loss, buccal and interradicular defects, sinus pneumatization reducing residual bone height to <6 mm, thin peri-implant soft-tissue phenotype) [Figures 1, 2]. |
| **Timeline** | 7. Historical and current information from this episode of care, organized as a timeline (figure or table is recommended). | Clinical Presentation, lines 102–178 – presented chronologically: extraction of #24/#25 → alveolar ridge preservation (lines 112–125) → 6-month healing/regenerated bone (lines 126–129) → crestal sinus floor elevation with simultaneous implant placement (lines 130–150) → 6-month osseointegration confirmation (lines 148–150) → staged soft-tissue augmentation at 4 months post-implant (lines 151–158) → ~3-month soft-tissue maturation (lines 159–164) → delayed-loading prosthetic phase (lines 165–170) → 2-year follow-up from crown delivery (lines 171–177). A summary timeline figure/table may still be added as supplementary material if requested by the journal. |
| **Diagnostic Assessment** | 8a. Diagnostic testing (e.g., laboratory, imaging, questionnaires). | Clinical Presentation, lines 90–97 – clinical periodontal examination and CBCT imaging [Figures 1, 2, 5]. |
|  | 8b. Diagnostic challenges (e.g., access to testing, financial, cultural). | None reported; standard diagnostic imaging and clinical assessment were available and performed (Clinical Presentation, lines 85–101). |
|  | 8c. Diagnosis (including other diagnoses considered). | Clinical Presentation, lines 89–101 – non-restorable, periodontally compromised premolars #24/#25 with advanced ridge atrophy and sinus pneumatization at site #26; short-implant alternative considered and discussed (Introduction, lines 46–54; Discussion, lines 211–225). |
|  | 8d. Prognosis (e.g., staging in oncology) where applicable. | Discussion, lines 211–261 – prognosis of hard- and soft-tissue outcomes discussed in relation to staged ARP, crestal sinus elevation, and timing of soft-tissue augmentation. |
| **Therapeutic Intervention** | 9a. Types of intervention (e.g., pharmacologic, surgical, preventive, self-care). | Clinical Presentation, lines 102–170 – extraction, alveolar ridge preservation (xenograft + resorbable collagen membrane), minimally invasive crestal sinus floor elevation, implant placement, connective tissue grafting, delayed-loading screw-retained metal-ceramic prosthetic restoration. |
|  | 9b. Administration of intervention (e.g., dosage, strength, duration). | Clinical Presentation, lines 119–170 – implant dimensions (4.5×11 mm, 4.5×10 mm; OSSTEM, Osstem Implant Co.), CAS-Kit transcrestal technique, hydraulic membrane elevation (≈3–4 mm lift), insertion torque of 35 Ncm (line 146), graft materials (Bio-Oss®, Bio-Gide®) and healing intervals specified (6 months post-ARP, line 127; 6 months post-implant, line 148–150; 4 months to soft-tissue grafting, line 151; ~3 months soft-tissue maturation, lines 159–164). |
|  | 9c. Changes in intervention (with rationale). | Clinical Presentation/Discussion, lines 226–230 – staged (rather than immediate) crestal sinus elevation with delayed implant placement was adopted due to severe vertical deficiency; lines 247–261 – soft-tissue augmentation was deliberately staged after osseointegration, with rationale explained. |
| **Follow-up and Outcomes** | 10a. Clinician- and patient-assessed outcomes (if available). | Clinical Presentation, lines 171–177 – 2-year follow-up (from crown delivery): stable osseointegration, healthy peri-implant soft tissue, well-contoured emergence profile, and patient-reported esthetic/functional satisfaction (also Abstract, lines 29–32) [Figure 13]. |
|  | 10b. Important follow-up diagnostic and other test results. | Clinical Presentation, lines 148–150 – radiographic follow-up at 6 months confirming osseointegration and stable crestal bone levels [Figure 10]; lines 171–174 – clinical and radiographic stability confirmed at 2 years. |
|  | 10c. Intervention adherence and tolerability (how was this assessed?). | Clinical Presentation, lines 171–177 – no adverse events, complications, or tolerability issues were noted throughout treatment and follow-up; assessed clinically and radiographically at each stage. |
|  | 10d. Adverse and unanticipated events. | Clinical Presentation, lines 172–174 – explicitly stated that no biological complications (mucositis, suppuration, radiolucency) or prosthetic complications (screw loosening, chipping, loss of retention) occurred during the follow-up period. |
| **Discussion** | 11a. Strengths and limitations of the management of this case. | Discussion, lines 179–270 – strengths of the staged, biologically guided protocol are discussed throughout; as a single-patient case report, generalizability is inherently limited. Note: References 37 and 38 (lines 403–408) are flagged in the manuscript as pending full bibliographic verification by the authors prior to resubmission – this should be resolved before final publication. |
|  | 11b. Discussion of the relevant medical literature. | Discussion, lines 179–261, full section – extensive review of alveolar ridge preservation, crestal sinus elevation, and peri-implant soft-tissue/phenotype literature, with 36 fully cited references and 2 references (37–38) pending verification (Reference list, lines 303–408). |
|  | 11c. The rationale for conclusions (including assessment of possible causes). | Discussion, lines 226–261 – rationale for staged crestal sinus elevation, delayed implant placement, and staged soft-tissue augmentation explained with supporting evidence. |
|  | 11d. The primary “take-away” lessons of this case (without over-generalizing). | Conclusion, lines 262–270 – a sequential, biologically guided staged strategy (ARP, minimally invasive crestal sinus elevation, delayed soft-tissue augmentation) is presented as a reliable framework for managing atrophic posterior maxillary sites. |
| **Patient Perspective** | 12. The patient should share their perspective or experience in a statement (where possible/applicable). | Patient-reported esthetic and functional satisfaction at the 2-year follow-up is noted (Abstract, lines 29–32; Clinical Presentation, lines 171–177). A direct first-person patient quotation is not included and may be added if the authors wish and the patient consents. |
| **Informed Consent** | 13. Did the patient give informed consent? Please provide if requested. | Declarations – Ethics Approval and Consent, lines 271–280 – written informed consent for the clinical procedures and for publication of anonymized clinical data and images was obtained from the patient; IRB review was waived per institutional policy for single, anonymized retrospective case reports; consent documentation available upon request. |

*Reference: Gagnier JJ, Kienle G, Altman DG, Moher D, Sox H, Riley D; CARE Group. The CARE guidelines: consensus-based clinical case reporting guideline development. BMJ Case Rep. 2013.*
